# Common housekeeping proteins are upregulated in colorectal adenocarcinoma and hepatocellular carcinoma, making the total protein a better "housekeeper"

**DOI:** 10.18632/oncotarget.11439

**Published:** 2016-08-20

**Authors:** Xiaowen Hu, Shujiao Du, Jiekai Yu, Xuhan Yang, Chao Yang, Daizhan Zhou, Qingyu Wang, Shengying Qin, Xiaomei Yan, Lin He, Dongmei Han, Chunling Wan

**Affiliations:** ^1^ Bio-X Institutes, Key Laboratory for The Genetics of Developmental and Neuropsychiatric Disorders, Ministry of Education, Shanghai Jiao Tong University, Shanghai, 200030, PR China; ^2^ Cancer Institute, Key Laboratory of Cancer Prevention and Intervention, The Second Affiliated Hospital, Zhejiang University, College of Medicine, Hangzhou, Zhejiang, 310009, PR China; ^3^ School of Life Science and Biotechnology, Shanghai Jiao Tong University, Shanghai, 200240, PR China; ^4^ Instrumental Analysis Center, Shanghai Jiao Tong University, Shanghai, 200240, PR China

**Keywords:** normalization, endogenous control, housekeeping protein, total protein amount, tumor

## Abstract

Housekeeping proteins are essential endogenous controls for normalization as they are expected to be stably expressed. However, the stability of the expression level of housekeeping proteins needs to be assessed considering various experimental conditions. Our study evaluated the degree of variability of 7 commonly used housekeeping proteins with regard to their potential utility as normalizers in 56 pairs of matched colorectal adenocarcinoma (CRC) tissue samples and 6 pairs of hepatocellular carcinoma (HCC) tissue samples using multiple reaction monitoring (MRM) and Western blot analyses. A comprehensive experimental design and strict statistical analysis revealed that the expression levels of these 7 housekeeping proteins were not as stable as expected and they all exhibited upregulations to varying degrees in both the CRC and the HCC tissue samples. Consequently, we verified that using the amount of total protein instead of that of an individual protein can serve as a preferable control for studies of protein expression that require normalization.

## INTRODUCTION

The endogenous control is a widely used concept in gene expression studies, as it is the foundation of accurate quantification. An inappropriate endogenous control often drastically affects the accuracy and reliability of the results and may even completely subvert the outcomes. Generally, the endogenous control employed for normalization should meet several strict criteria. The control should exhibit constitutive, nonregulated and stable expression, regardless of tissue types or experimental designs. Additionally, the control should be expressed at a certain level which could easily reach the limit of detection.

Housekeeping genes, which mainly function in cellular maintenance, are commonly used as endogenous controls as they are often considered to be adequately and stably expressed [1]. However, an ideal gene for this purpose does not actually exist. Cells are exposed to rapidly changing microenvironments as a result of various changes in metabolic conditions. To survive these stresses, cells must adopt various strategies to increase their adaptability to the rapidly changing microenvironments [2]. These subtle adjustments may affect both the active genes, which are sensitive to local environments, and the housekeeping genes, which maintain vital functions.

Commonly used reference genes, such as ACTB, GAPDH, TUBB, 18S-rRNA, HPRT1 and UBC, have been investigated on the mRNA level in various studies in different tissues and organisms using quantitative real-time polymerase chain reaction (RT-PCR) [2–6]. All of these studies concluded that the expression levels of housekeeping genes are not as stable as we used to expect and there was no universally accepted “the best reference gene” for normalization [5], demonstrating the need for a comprehensive evaluation and selection of an appropriate reference gene before conducting gene expression studies. This need led to the creation of a series of software programs, such as geNORM [7], NormFinder [8] and BestKeeper [9], to evaluate the stability of gene expression with the goal of suggesting a gene with a relatively consistent expression level based on certain algorithms [3–6, 10, 11].

Current studies evaluating the stability of housekeeping gene expression primarily focused on mRNA levels [2–4, 6, 10, 11]. Studies on endogenous protein controls remain urgently required. Some researchers realized the issue and examined the degree of variability of several proteins using Western blots [12–15]. Similar results were obtained that the commonly used housekeeping proteins such as ACTB and GAPDH changed their expression levels on certain circumstances and could not be employed as reliable controls without a comprehensive evaluation [14, 15]. However, it is generally difficult to comprehensively evaluate and select an appropriate reference gene owing to various limiting factors. To our knowledge, the present study is the first systematic quantitative research that evaluated the stability of 7 commonly used housekeeping proteins with regard to their potential utility as normalizers using two different technical platforms in large-scale sample sets of matched tumor and the adjacent non-cancerous tissues from patients diagnosed with colorectal adenocarcinoma (CRC) or hepatocellular carcinoma (HCC). To investigate the subtle differences in the tumor tissues and their matched non-cancerous tissues, multiple reaction monitoring (MRM) performed by mass spectrometry screening was adopted due to its excellent resolution and high throughput in quantitative proteomic researches; the traditional approach of immunoblotting was subsequently employed to validate the results. Our study could serve as a referrible guidance on the normalization of protein expression in tumors.

## RESULTS

### MRM quantitative proteomics revealed the elevated expression of housekeeping proteins in CRC tumor tissues

**Figure 1:**
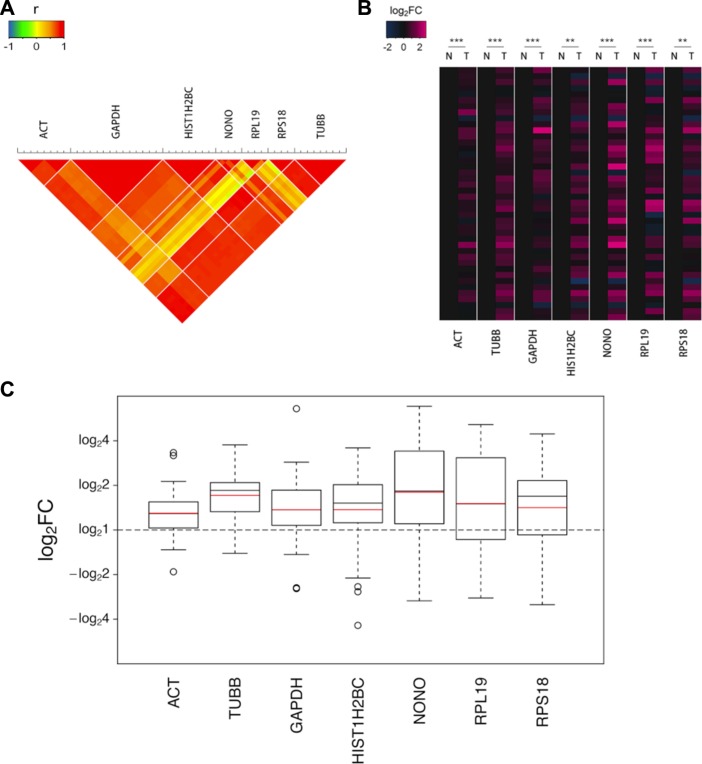
The upregulations of the 7 candidate housekeeping proteins in tumor tissues of the discovery sample set (CRC) quantified using the MRM assay (**A**) The correlation (Pearson's test) in a set of monitored transitions fragmented from peptides that were enzymatically digested from the same protein. (**B**) The heat map of the log_2_FCs of the 42 pairs of valid data. The values for the non-cancerous tissues were assigned as zeros to obviously identify the trends in the changes in the tumor tissues. The statistical significance: **p < 0.01; ***p < 0.001. (**C**) The boxplots of the log_2_FCs of the 42 pairs of valid data. The boxes and whiskers indicate the minimum value, the 25th percentile, the mean (red), the median (black), the 75th percentile and the maximum value.

A strong correlation (Pearson's test) was observed in a set of monitored transitions fragmented from peptides that were enzymatically digested from a single protein (Figure 1A). As shown in the heat map of log_2_FC (Figure 1B), the seven selected proteins all displayed upregulations in most of the tumor tissue samples to varying degrees. Marked differences were noted between the tumor and non-cancerous tissues for all 7 housekeeping proteins, three of which exhibited sharp increases in the tumor with changes of greater than 1.5-fold (Figure 1C).

The three most commonly used endogenous controls in protein expression assays, ACTB, TUBB and GAPDH, all displayed significant elevations in tumor samples, indicating that these proteins may not be as reliable as expected. In particular, TUBB exhibited a remarkable increase with a fold change (FC) up to 1.71. ACTB, which is the most commonly used endogenous control in various experiments, is one of the two isoforms of cytoplasmic actin, the other of which is ACTG1. ACTB and ACTG1 are highly homologous that they share the identical sequence in a percentage of up to 98.9% and only 4 amino acids in the N termini of the proteins differ. We failed to monitor their unique sequences respectively in MRM analysis due to the preference of mass spectrometry screening. Therefore, in this section, ACT refers to cytoplasmic actin, which encompasses both ACTB and ACTG1. Although ACT is widely considered to be the most consistently expressed gene in different samples and conditions, we observed that the level of ACT was considerably elevated by 1.30-fold in the tumor tissues in our study. GAPDH, another commonly used control protein, exhibited an even higher FC of 1.37.

In addition to the remarkable differences in the geometric means of the FCs, the high degree of dispersion is also worth noticing, as shown by the height of the boxes and the long whiskers in the boxplots (Figure 1C). These indicate the considerable interindividual variation that cannot be ignored in human beings, which is another limit of using these proteins as endogenous controls.

### Western blot assays of the 7 candidate housekeeping proteins in an independent CRC sample set validated the elevated expression in tumor tissues

**Figure 2:**
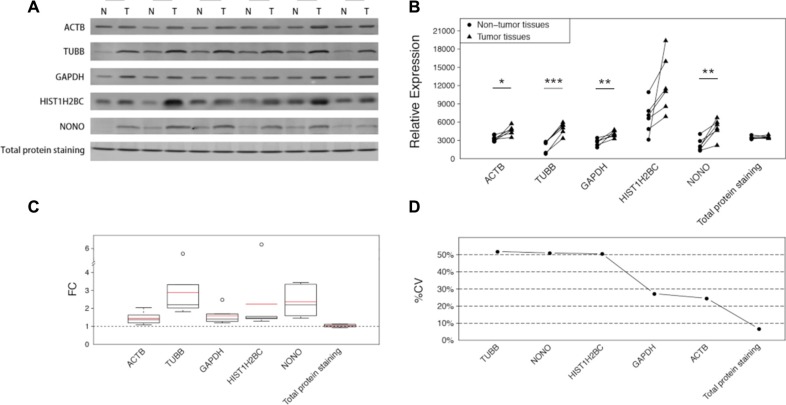
The differences of the 5 candidate housekeeping proteins and total protein staining between tumor and non-cancerous tissues in the validation sample set (CRC) (**A**) Western blot analyses of ACTB, TUBB, GAPDH, HIST1H2BC, and NONO, and the total protein staining with SYPRO Ruby in an SDS-PAGE stacking gel. (**B**) The intensities of the bands in (A) quantified using ImageJ software. The statistical significance: *p < 0.05; **p < 0.01; ***p < 0.001. (**C**) The fold changes of the 5 candidate housekeeping proteins and the total protein staining. The boxes and whiskers indicate the minimum value, the 25th percentile, the mean (red), the median (black), the 75th percentile and the maximum value. (**D**) The coefficients of variation of the 5 candidate housekeeping proteins and total protein staining.

Western blot analyses were employed to verify the results of the MRM analysis in another CRC sample set, with the exception of protein RPL19 due to the lack of a detectable antibody and protein RPS18, which failed to reach the limit of detection in CRC samples. In addition, the total protein was examined by SYPRO Ruby staining in an SDS-PAGE stacking gel. The differences are easily illustrated in Figure 2A. Similarly to those observed in the MRM analysis, all the 5 proteins were upregulated to varying degrees in the tumor tissues. ACTB and GAPDH exhibited discernable differences with average FCs of 1.45 and 1.58 respectively (Figure 2C). The other three proteins exhibited larger changes which were easily distinguished, and TUBB displayed the largest change, with an average FC of 2.88. HIST1H2BC, the core component of the nucleosome, is typically used as a nucleolar control. Although the statistical significance of HIST1H2BC was not so strong owing to the noticeable variances among different tissue pairs, the difference within each pair was apparent (Figure 2A) and the largest change reached up to 6.22-fold (Figure 2C). Compared with these reference proteins, the total protein amounts, as interpreted by SYPRO Ruby staining, showed a small, barely discernable difference of 1.04-fold.

In addition to the remarkable FCs, which indicated the marked differences between the tumor tissues and the corresponding non-cancerous tissues, the large variations among all the 12 samples were also obvious (Figure 2B). To clearly identify the variations, we calculated the coefficient of variation (%CV) for each protein among all of the 12 specimens (Figure 2D). TUBB exhibited the largest %CV (51.87%), followed by NONO (50.99%) and HIST1H2BC (50.49%). Although GAPDH and ACTB performed better than the other housekeeping proteins, their %CVs were still greater than 20%, which is a widely accepted criterion for stability. However, the total protein, as stained with SYPRO Ruby, exhibited excellent consistency, with a %CV of 6.74%.

### The instability of the expression of housekeeping proteins also occurs in HCC sample set

**Figure 3:**
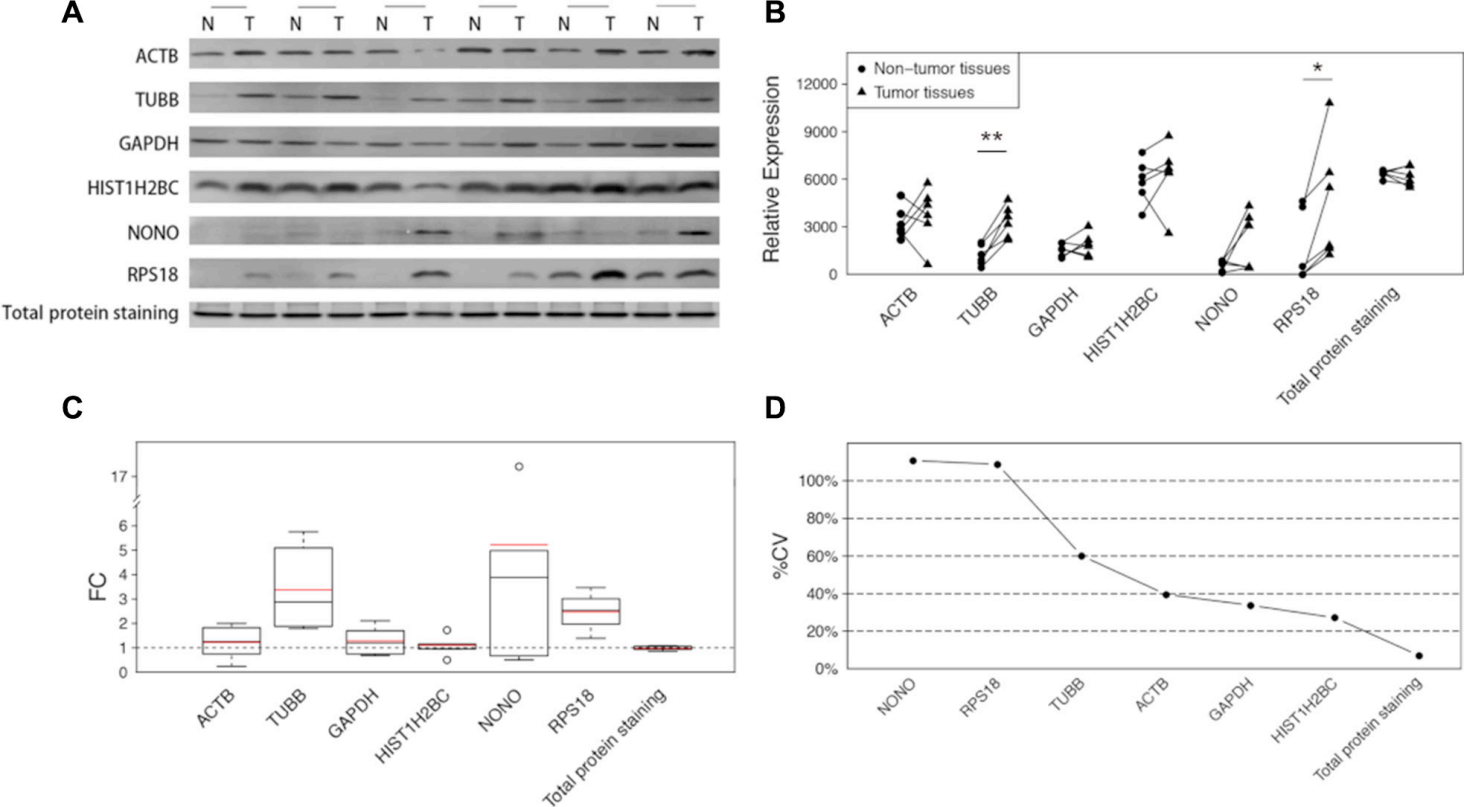
The differences of the 6 candidate housekeeping proteins and total protein staining between tumor and non-cancerous tissues in the extension sample set (HCC) (**A**) Western blot analyses of ACTB, TUBB, GAPDH, HIST1H2BC, NONO, and RPS18, and the total protein staining with SYPRO Ruby in an SDS-PAGE stacking gel. (**B**) The intensities of the bands in (A) quantified using ImageJ software. The statistical significance: *p < 0.05; **p < 0.01. (**C**) The fold changes in 6 candidate housekeeping proteins and total protein staining. The boxes and whiskers indicate the minimum value, the 25th percentile, the mean (red), the median (black), the 75th percentile and the maximum value. (**D**) The coefficients of variation of 6 candidate housekeeping proteins and total protein staining.

Our study was extended to a set of paired hepatocellular carcinoma samples to explore whether the inconstancy of the housekeeping protein levels was also present in other tumor types. Western blot analyses were again employed to analyze the performance of each specimen. Although the average FCs were not as large as in the CRC samples (Figure 3C), the inconsistent expressions of all the 6 detected housekeeping proteins were clearly noted in the sample set (Figure 3A). Some housekeeping proteins showed both increases and decreases in tumor tissues (Figure 3A–3B), which decreased the statistical power of the overall evaluations (Figure 3B). The large variations are illustrated in the calculation of %CVs, and some proteins exhibited %CVs of greater than 100% (Figure 3D). Nevertheless, the total protein, as demonstrated by SYPRO Ruby staining, showed constant sample amounts with a negligible FC (−1.03) and a small %CV (7.03%). (Figure 3A–3D).

### Subtle differences can be concealed in Western blots

**Figure 4:**
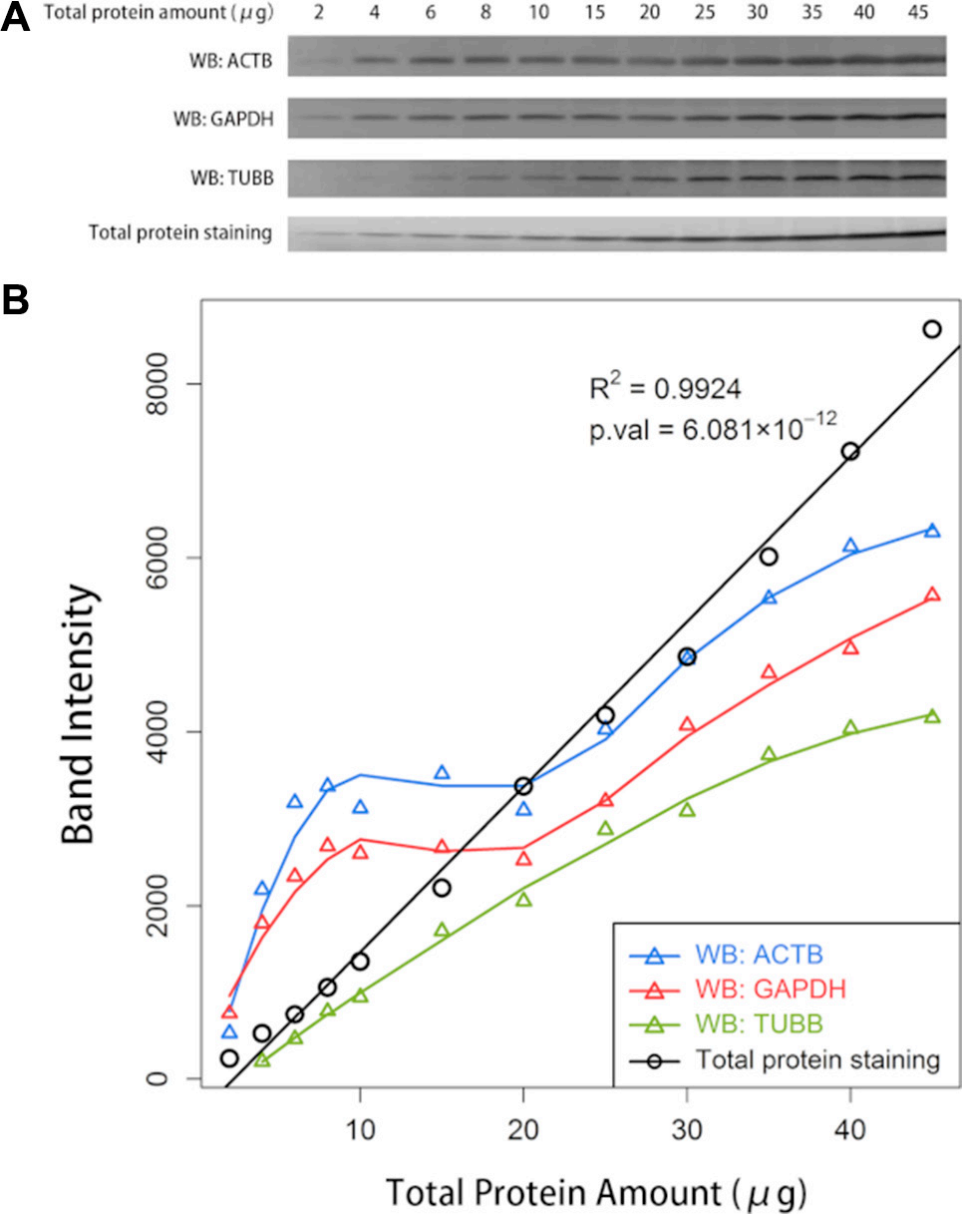
The comparison between the Western blot analysis of an individual protein and the total protein staining (**A**) Western blot (WB) analyses of ACTB, GAPDH, TUBB, and the total protein staining with SYPRO Ruby with serial gradient amounts of total protein loaded on the gels. (**B**) The relationship between the quantified band intensities and the actual amounts of the total protein loaded on the gels. The band intensities were quantified using ImageJ software.

We noticed an interesting phenomenon that differences were attenuated when large amounts of protein were loaded for Western blotting. Subtle differences would even disappear with large loading amounts, particularly for proteins with high abundance. To verify our assumption, an additional Western blot analysis was conducted with serial gradient loading amounts of total protein. The difference was indiscernible at certain intervals of loading amounts in the Western blots of ACTB, GAPDH and TUBB (Figure 4A). The quantification of the bands for both ACTB and GAPDH revealed a plateau in the range of approximately 8 to 20 μg of total protein. The curve increased sharply but not linearly to the loading amounts in the range of greater than 20 μg. Although no plateau occurred for TUBB, the band intensities of TUBB increased nonlinearly to the total protein amounts. However, the intensities of total protein staining with SYPRO Ruby revealed an excellent linearity with loading amounts, with a coefficient of determination (*R*^2^) of up to 0.9924 (Figure 4B).

## DISCUSSION

### Housekeeping proteins do not keep house so well as we expected in CRC and HCC

Endogenous controls are the foundation for accurate quantification. However, it has been a controversial issue ever since the first adoption of the so-called “reference genes” used as internal controls. In recent years, an increasing number of studies have illustrated that the expressions of these genes were also regulated in certain circumstances in response to the various stresses of rapidly changing microenvironments [3–6, 15]. This phenomenon is particularly remarkable in tissue specimens of some diseases, [12, 16–18] particularly in tumor tissues [5, 6, 11, 12, 19]. Tumors are peculiar as they are in an aggressive state of proliferation. Various signaling pathways in tumors are significantly disrupted and the metabolism is reprogrammed to a great extent [20, 21]. The increasing demand for building blocks compels tumor cells to adjust the limited available energy to promote survival and growth. Considering the predisposition to proliferation in tumors, the remarkable upregulations of housekeeping proteins exhibited in our study could be partially explained as that housekeeping genes, which are vital to sustaining basal living, tend to be actively expressed. By contrast, genes that are dispensable for survival may be suppressed in tumors.

To reliably and accurately evaluate the potential utility of 7 candidate housekeeping proteins as normalizers, we elaborately designed and conducted a comprehensive study. We recruited matched pairs of tumor tissues and the adjacent non-cancerous tissues instead of unmatched specimens to objectively determine the interindividual variation. The large sample scale reduced the impact of the individual effects of some particular cases and provided more power for the statistical analysis. MRM, a high-throughput targeted quantitative proteomics approach using liquid chromatography-tandem mass spectrometry (LC-MS/MS) with fine resolution, was used in the discovery experiment to distinguish the subtle differences in the candidate reference protein expression. Western blot analysis was employed in the validation experiment in an independent set of CRC specimens to verify the results of the MRM analysis. ACT, GAPDH and TUBB, the three most commonly used reference proteins, all exhibited marked elevations in tumor samples. Notably, GAPDH is one of the metabolic enzymes that participate in glycolysis, which is widely approved to be highly activated in tumors during the metabolic change called the Warburg effect or aerobic glycolysis [20, 21]. Therefore, it is understandable that GAPDH displayed an increasing trend in tumor tissues, which has been proved in several studies [22]. This also provides an evidence that there are no ideal proteins for normalization. Similar conclusions can be drawn from the extension experiment using the HCC sample set. Although the statistical significances were not as strong as in the CRC samples, the conclusion that housekeeping proteins were variably expressed remained valid in the HCC sample set, indicating that normalization based on certain housekeeping proteins may cause errors to the comparison of the real difference of target proteins. Additionally, the large variations, including the variations in the different FCs (Figures 2C and 3C) and the variations of the different expression levels in the same pathological class of tumor tissues or non-cancerous tissues (Figures 2B and 3B), indicate significant interindividual variations in the CRC samples and the HCC samples, which points out that the use of any one of these housekeeping proteins as a reference would be inappropriate.

### Western blot assays may not be appropriate for the detection of subtle differences

We noticed that the differences between cases were attenuated when Western blotting was performed. Subtle differences which could be easily distinguished by LC-MS/MS analysis were minimal or even disappeared in Western blots. What's worse, the signals lost their linearity with the loading amounts in Western blots. However, the amounts of total protein were perfectly presented by the SYPRO Ruby staining, and the signals exhibited an excellent linearity with the loading amounts. We blamed this discrepancy on the difference in the resolving capability and the dynamic range of different approaches, which are essential elements for detecting real differences. The SYPRO Ruby family of fluorescent dyes has a high sensitivity and a broad dynamic linear range. Studies revealed that the sensitivity of this type of staining could reach up to approximately 50 ng per protein band [23, 24]. While in the process of Western blots, signals are cascade-amplified step by step and they will easily reach saturating intensities, between which they lose their linearity with the actual loading amounts [25]. The phenomenon became severer when the problem came to proteins with high abundances, such as ACTB and GAPDH. Although TUBB performed better, the signal intensities were still not linear correlated to the actual loading amounts (Figure 4B). And the limited linear range of each protein could be found at different intervals of loading amounts due to their different abundances [12]. What's more, the saturating intensity range differs on an antibody-dependent way when different antibodies were used to detect a same protein [14]. Thus, real differences would be falsely exhibited and subtle differences may be concealed with the poor resolution or in an inappropriate dynamic range, which may mislead us to conclude that these proteins were stably expressed. When we shifted to approaches with higher sensitivity, better resolution and wider linear dynamic range, such as mass spectrometry screening, the fine details could be easily presented.

### The measurement of the total protein amount instead of the amount of an individual protein is a preferable choice for studies of protein expression that require normalization

A normalization control is used to correct the uneven efficiencies of various experimental procedures, in order that we can compare the real difference of target molecules on the basis of the same total amount. Housekeeping proteins are employed as normalizers for decades as they were considered to be expressed so stably that the amount of total protein can be reflected by that of an individual housekeeping protein. However, the premise is untenable in many circumstances as discussed in our work and previously reported in many studies [12–15]. In this case, an alternative endogenous control is in need to eliminate the errors from sample amounts. The direct measurement of total protein amounts exhibited an excellent performance as we tested and discussed in our study. The amount of total protein can be measured by different methods in different technical approaches.

In Western blot assays, the total protein can be presented with staining in a paralleled electrophoretic gel. Proteins will be stained by dyes in an unselective way, thus the quantification of staining is a good reflection of the real sample amounts, which makes the normalization to the quantification of total protein staining reasonable to eliminate the errors from sample amounts. As illustrated in Figures 4A and 4B, the band intensities of total protein staining with SYPRO Ruby exhibited a strong linear correlation with the actual loading amounts, offering a preferable choice to present the total protein amount and serve as a good normalizer. A growing number of researchers have already realized this issue and adopted total protein staining instead of the detection of the immunoblot signal of an individual housekeeping protein as the reference for normalization in Western blot analysis [12, 13, 26–28]. However, they have primarily adopted the method of staining the proteins in SDS-PAGE separating gels, in which proteins would be separated into a series of bands depending on their molecular weights. We recommend to stain the total protein in an SDS-PAGE stacking gel with no separating gel below, as all of the proteins are stacked into a single band regardless of their molecular weights, which supplies an intuitive way to distinguish the differences and conveniently facilitate the quantification with some image processing software programs. Varied methods of staining are available when detecting the total protein, such as SYPRO Ruby staining, Coomassie Brilliant Blue staining, Reversible Ponceau S staining or using staining-free gels instead [27].

In the field of high-throughput quantitative proteomic studies performed with mass spectrometry screening, normalization using the sum of the total peptide ion signals (for label-free quantification) or total reporter ion signals (for labeled quantification) during data analysis has gained increasing acceptance, and some software programs have already developed some optimized algorithms based on the total signals to rescale the data [29].

In conclusion, housekeeping proteins do not “keep house” so well as we used to expect regarding the upregulations of the candidate proteins in colorectal adenocarcinoma and hepatocellular carcinoma in our study. To select an appropriate normalization reference, the amount of total protein rather than that of an individual protein is recommended as a better control for studies of protein expression.

## MATERIALS AND METHODS

### Patients and samples

Colorectal adenocarcinoma tumor tissues and matched adjacent non-cancerous tissues were obtained from 56 patients (50 pairs for the discovery experiment and 6 pairs for the validation experiment, see Table 1) from the Second Affiliated Hospital, Zhejiang University, College of Medicine. Paired hepatocellular carcinoma tumor and non-cancerous tissues were collected from 6 patients in the Shanghai Oriental Hepatic Hospital and used in the extension experiment. Informed consent was obtained from all patients, and the project was approved by the local ethics committee.

**Table 1:** Summary of patient demographics and tumor characteristics

			CRC Discovery Set (MRM)	CRC Validation Set (Western Blot)	HCC Extension Set (Western Blot)
			(*n* = 50)	(*n* = 6)	(*n* = 6)
Age (Mean ± SD)			61.2 ± 11.5	60.0 ± 8.6	55.3 ± 7.8
Gender (Male:Female)			27:23	5:1	5:1
	Stage I		6	2	0
	Stage II		13	3	0
		A	1		
TNM Stage	Stage III	B	9	0	6
		C	6		
	Stage IV		3	1	0
	n/a		12	0	0

### Protein extraction and preparation

The tissue samples were minced with surgical scissors in sample buffer (7 M urea, 2 M thiourea, 4% CHAPS, 65 mM dithiothreitol, and 40 mM Tris base) and then homogenized with a Scientz-48 homogenizer (SCIENTZ, Ningbo, Zhejiang, China). Soluble proteins were obtained by centrifugation, and protein concentrations were measured with the Bradford Protein Assay (BIO-RAD, Hercules, CA, USA). For liquid chromatography-tandem mass spectrometry (LC-MS/MS) analysis, soluble proteins were precipitated with ethanol: acetone: acetic acid (v:v:v = 50:50:1) and resolved with 6 M guanidine hydrochloride and 50 mM ammonium bicarbonate. The proteins were reduced with 20 mM dithiothreitol for 1 h at 56°C, and the reduced lysate was alkylated with 90 mM iodoacetamide for 40 min at room temperature in the dark. The lysate buffer was then exchanged into 50 mM ammonium bicarbonate using a Vivacon 500 concentrator (Sartorius Stedim Biotech, Goettingen, Germany) to protect the tryptic working environment in the next step from the high salt concentration. The proteins were digested overnight with trypsin (Promega, Madison, WI, USA) at a trypsin:substrate ratio of 1:100. The digested peptide lysates were acidified with formic acid and dried at room temperature under vacuum with a ConcentratorPlus (Eppendorf, Hamburg, Germany).

### Selection of candidate reference proteins and their unique peptides

Seven commonly used reference genes were selected to assess the stability of their expression (Table 2). Unique peptides of each protein were selected with the guidance of PeptideAtlas (http://www.peptideatlas.org). The number of monitored transitions fragmented from the unique peptides of each protein ranged from 4 to 14.

**Table 2:** Panel of 7 candidate housekeeping proteins

Gene Symbol	Protein Name	Cellular Function	Number of monitored transitions
ACTB	Beta-actin, cytoplasmic	Cytoskeletal structual protein	8
GAPDH	Glyceraldehyde-3-phosphate dehydrogenase	Glycolytic enzyme	14
HIST1H2BC	Histone H2B type 1-C/E/F/G/I	Core component of nucleasome	8
NONO	NonO protein	DNA and RNA binding protein	4
RPL19	60S ribosomal protein L19	Structural constituent of ribosome	4
RPS18	40S ribosomal protein S18	Structural constituent of ribosome	4
TUBB	Tubulin beta chain	Major constituent of microtubules	8

### LC-MS/MS analysis

A total of 50 transitions for 7 housekeeping proteins were subjected to multiple reaction monitoring (MRM) on a QTRAP 5500 (AB SCIEX, Framingham, MA, USA) after the optimization of assay conditions. The raw data were analyzed with MultiQuant software (version 2.1.1, AB SCIEX, Framingham, MA, USA). All quantification was performed at the transition level, and the peak of each transition was manually verified and optimized for accurate quantification.

### Quality control

We pooled all of the 100 test samples at equimolar concentrations as a quality control sample (QC). The QC sample was injected into the LC-MS/MS apparatus after every 10 injections of the test samples to evaluate the performance of the equipment.

### Statistical analysis of the MRM data

One pair of matched samples was excluded from the subsequent analysis owing to ambiguous clinical information. The coefficient of variation (%CV) for the 11 QC injections for each transition was calculated separately, and all the 50 transitions exhibited good reproducibility, with %CVs less than 15%. Of these transitions, 96% (48 out of 50) displayed %CVs less than 10%. Missing value imputation was performed using the k-NearestNeighbor (kNN) algorithm. Three samples were removed during the imputation as more than 60% of their values were missing, thereupon their matched samples were excluded as well. Log_2_ transformation was performed on the raw intensity, which refers to the peak area of the transition signal in LC-MS/MS analysis. The Kolmogorov–Smirnov test was performed after the log_2_ transformation to ensure the data fit a normal distribution. As a significant correlation (Pearson's test) was observed in a set of monitored transitions fragmented from peptides that had been enzymatically digested from a single protein (Figure 1A), we assumed that the expression pattern of an individual protein can be represented by any one of its individual transitions. Therefore, for each protein, we chose the transition with the highest intensity which fit the normal distribution in both the tumor class and non-cancerous sample class as the representative transition. A principal component analysis of the 7 representative transitions for the 7 proteins was performed and identified 4 sample outliers, which should be excluded to minimize the effect of extreme values under the guidance of the first and second principal components, which explained 67.3 and 12.4% of the variability respectively (79.7% in total). After strict quality controls at the statistical level, the data from 42 pairs of tissues remained valid and were used for the subsequent analysis. Paired *t*-test was performed between the tumor tissues and the matched non-cancerous tissues. A marked difference was defined with a difference greater than 1.2-fold in the geometric mean of the fold change (FC), together with a *p*-value less than 0.05, which indicated statistical significance. All of the statistical analyses were performed using the R environment.

### Sodium dodecyl sulfate polyacrylamide gel electrophoresis (SDS-PAGE) and Western blotting

The proteins were size-fractionated by electrophoresis on 15% SDS-PAGE gels. Western blotting was performed as previously described [30]. Antibodies specific for the following proteins were used: ACTB (AA128, Beyotime), TUBB (#2128, Cell Signaling Technology), GAPDH (AG019, Beyotime), HIST1H2BC (ab52599, Abcam), NONO (ab109511, Abcam), and RPS18 (ab91293, Abcam). Different amounts of total protein were loaded for different immunoblots to well distinguish subtle differences on the premise of reaching the limit of detection. On the other hand, the total protein were stacked into a single band on a 5% SDS-PAGE stacking gel with no separating gels below and subsequently stained with SYPRO Ruby (#170-3125, BIO-RAD). The band intensities were quantified using ImageJ software (National Institutes of Health).

### Statistical analysis of the data of Western blot assays and SYPRO Ruby stainings

The band intensities of all the Western blot assays and SYPRO Ruby stainings fit normal distributions in both the tumor class and the non-cancerous sample class, which were examined by Kolmogorov–Smirnov tests separately. The paired *t*-tests were performed between the tumor class and non-cancerous class to the Western blots for each protein, together with the total protein stainings with SYPRO Ruby respectively. All of the statistical analyses were performed using the R environment.
